# Toll-like receptor 4 deficiency ameliorates β2-microglobulin induced age-related cognition decline due to neuroinflammation in mice

**DOI:** 10.1186/s13041-020-0559-8

**Published:** 2020-02-14

**Authors:** Qi Zhong, Yufeng Zou, Hongchao Liu, Ting Chen, Feng Zheng, Yifei Huang, Chang Chen, Zongze Zhang

**Affiliations:** 1 Department of Anesthesiology, Zhongnan Hospital, Wuhan University, East Lake Road, Wuhan, 430071 Hubei China; 2 Department of Anesthesiology, Maternal and Child Hospital of Hubei Province, Wuluo Road, Wuhan, 430071 Hubei China

**Keywords:** TLR4, B2M, Age-related cognitive decline, Neuroinflammation, Apoptosis, Neurogenesis, Synaptic function

## Abstract

Toll-like receptor 4 (TLR4) is a crucial receptor in neuroinflammation and apoptotic neuronal death, and increasing evidences indicated that β2-microglobulin (B2M) is thought to be a major contributor to age-related cognitive decline. In present study, we designed to investigate the effects of TLR4 on B2M-induced age-related cognitive decline. Wild-type (WT) C57BL/6, TLR4 knockout (TLR4 -KO) mice and hippocampal neurons from the two type mice were respectively divided into two groups: (1) Veh group; (2) B2M-treated group. The behavioral responses of mice were measured using Morris Water Maze. Hippocampal neurogenesis and neuronal damage, inflammatory response, apoptosis, synaptic proteins and neurotrophic factors, and TLR4/MyD88/NF-κB signaling pathway proteins were examined using molecular biological or histopathological methods. The results showed that WT mice received B2M in the DG exhibited age-related cognitive declines, increased TLR4 mRNA expression and high levels of interleukin-1β (IL-1β), tumor necrosis factor-alpha (TNF-α) and apoptotic neuronal death in the hippocampus, which were partially attenuated in TLR4-KO mice. Moreover, in absence of TLR4, B2M treatment improved hippocampus neurogenesis and increased synaptic related proteins. Our cell experiments further demonstrated that deletion of TLR4 could significantly increase synaptic related protein, decrease neuroinflammatory fators, inhibited apoptotic neuronal death, and regulated MyD88/NF-κB signal pathway after B2M treatment. In summary, our results support the TLR4 contributes to B2M-induced age-related cognitive decline due to neuroinflammation and apoptosis through TLR4/MyD88/NF-κB signaling pathway via a modulation of hippocampal neurogenesis and synaptic function. This may provide an important neuroprotective mechanism for improving age-related cognitive decline.

## Introduction

Literatures have noted that advanced age is the highest risk factor for physiological decline in both physically and psychologically, including decreased neurogenesis and cognitive impairment [1–3]. Seriously, these mild physiological decline have been shown to increase risk to develop dementia later in life [4]. Therefore, deeper understanding of the physiological mechanism in aging-related cognitive declines is necessary for counteracting vulnerability to cognitive dysfunction.

Heterochronic parabiosis evidences have demonstrated an age-dependent bi-directionality in the influence of the systemic environment indicating pro-aging factors in old blood drive aging. It has also been proposed that alleviating the effect of pro-aging factors may provide an effective approach to rejuvenate aging phenotypes [5, 6]. Therefore, a subset of blood-borne immune-related factors identified as potential pro-aging factors played an important role in age-related cognitive dysfunction.

β2-microglobulin (B2M), a light chain of major histocompatibility complex class I (MHC I) [7], is a circulating factor that can promote age-related cognitive dysfunction in the adult hippocampus with an age-dependent manner by its inflammation and immune function [8]. Increased systemic levels of B2M have also contributed to impairs cognitive function in young mice. Considering the association between B2M levels and cognitive decline, B2M has been identified as a potential pro-aging factor which was implicated in age-related cognitive and regenerative impairments in the adult brain. However, how B2M mediated age-related impairments in the adult brain has not been fully investigated.

Toll-like receptor 4 (TLR4) is a type of pattern recognition receptor, and is involved in innate and adaptive immunity by producing pro-inflammation cytokines, chemokines and apoptosis [9] . Studies have reported that TLR4 in the central nervous system (CNS) involved in memory, learning impairment [10] and cognitive decline [11]. Recently, TLR4 was also identified as a modulator of neurogenesis in the brain during injuries, cerebral ischemia, and cognitive decline [12], and it is recognized to contribute to neuroplasticity [13]. Studies demonstrated that cognitive dysfunction was alleviated possibly by inhibiting hippocampal TLR4 activation in aged rats [14]. Moreover, the improvement of cognitive deficits was accompanied by the attenuation of inflammatory injury and apoptosis via TLR4 activation and its downstream signaling pathways [15, 16]. However, whether TLR4 contribute to β2-microglobulin (B2M)-induced age-related cognition decline in mice is unknown.

In this study, we hypothesized that neuroinflammation and apoptosis triggered by TLR4 may contribute to B2M-induced cognitive dysfunction. Using B2M-induced cognitive dysfunction model of wild-type (WT), TLR4 knockout (KO) mice and hippocampal neurons, we aimed to investigate the role of TLR4 in age-related learning and memory deficits. Thus, we tested the cognitive function of B2M-handled mice, and assessed the hippocampal structure changes, hippocmapal neurogenesis, synaptic protein, apotosis, as well as the expression of TLR4 mRNA and the levels of IL-1β, TNF-α, NGF and BDNF.

## Results

### TLR4 elimination prevented cognitive dysfunction following B2M treatment

Previous study showed that exogenous B2M injected locally in the hippocampus impairs hippocampus-dependent cognitive function in WT mice. However, it is not clear whether the same treatment affects the behavior of TLR4-KO mice. The evidence demonstrated the role of TLR4 in learning and memory function of Alzheimer’s disease (AD), we wondered if TLR4 affected B2M-induced cognition impairment. Therefore, we tested the effect of B2M, administrated in hippocampus, on cognitive behaviors in WT mice and TLR4-KO mice by Morris water maze test.

**Fig. 1:**
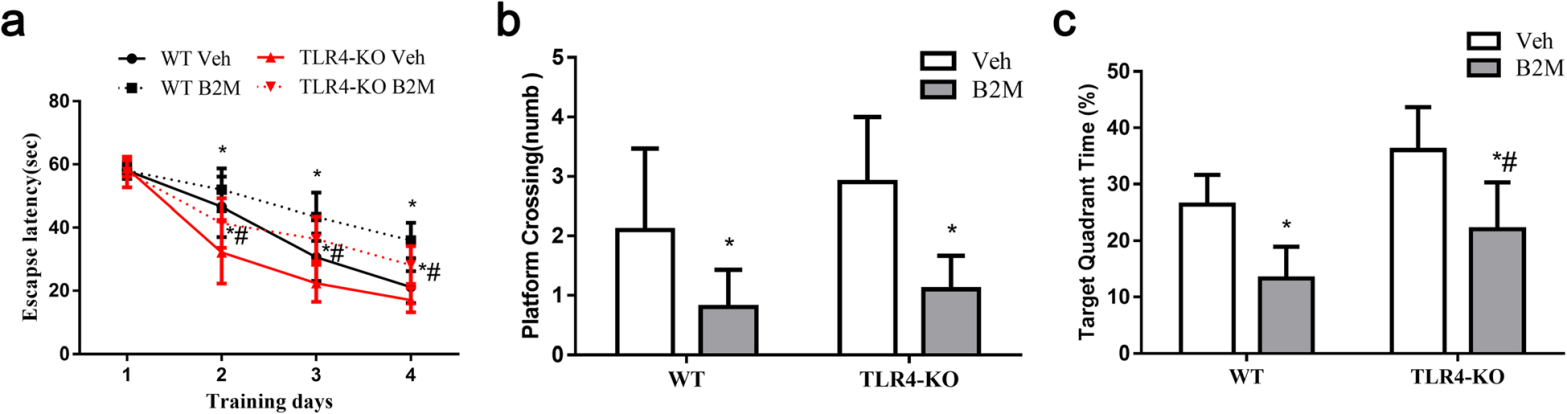
TLR4 elimination prevented cognitive dysfunction following B2M treatment. All groups of mice were trained in Morris water maze (MWM) test 23 days after B2M or vehicle treatment (*n* = 10/subgroup). TLR4 elimination attenuated spatial learning and memory dysfunction caused by B2M. The escape latency measured as mean time (**a**) were detected within 4 consecutive days. On the 28th day after treatment, the probe trail was conducted to record the platform crossing times (**b**) and mean percentage of time in the target quadrant (**c**). The data were analyzed using two-way ANOVAs used Tukey’s multiple comparisons test. Values are presented as the means ± SD. Significant differences are expressed as follows: ^*^p < 0.05 vs. Veh group, ^#^*p* < 0.05 vs. B2M group for WT mice. MWM = Morris water maze, WT = wild type C57BL/6, TLR4-KO = TLR4 knockout

With the training for 4 consecutive days, all groups of mice improved in the ability to reach the goal platform throughout the training trials, meaning a learning process in the test. As shown in Fig. 1a, B2M significantly impaired spatial learning ability in the WT mice (day3: 42.9 ± 3.9 for B2M group vs. 31.6 ± 2.8 for Veh group, *p =* 0.0014; day4: 34.6 ± 3.1 for B2M group vs. 21.2 ± 1.8 for Veh group, *p* < 0.0001) and TLR4-KO mice (day2: 42.4 ± 2.6 for B2M group vs. 28.8 ± 5.6 for Veh group, *p* = 0.0308; day3: 38.4 ± 2.6 for B2M group vs. 20.5 ± 2.1 for Veh group, *p =* 0.0001; day4: 28.1 ± 2.4 B2M group vs. 15.5 ± 1.7 for Veh group, *p =* 0.0001). Moreover, TLR4 elimination alleviated B2M-induced learning impairment (day2: 42.4 ± 2.6 for TLR4-KO mice vs. 50.4 ± 2.2 for WT mice, *p =* 0.0048; day3: 38.4 ± 2.6 for TLR4-KO mice vs. 42.9 ± 3.9 for WT mice, *p =* 0.0497; day4: 28.1 ± 2.4 for TLR4-KO mice vs. 34.6 ± 3.1 for WT mice, *p =* 0.0078).

Probe trials conducted 24 h after training on day 28 after B2M treatment. As shown in Fig. 1b and c, B2M treated group showed less platform crossing times and less time in the target zone compared with Veh group (platform crossing: WT mice: 1.0 ± 0.8 for B2M group vs. 2.6 ± 1.1 for Veh group, *p* = 0.0139; TLR4-KO mice: 1.1 ± 0.6 for B2M group vs. 3.1 ± 0.8 for Veh group, *P* = 0.0002; time in the target zone: WT mice: 12.9 ± 3.7 for B2M group vs. 26.7 ± 5.5 for Veh group, *p* < 0.0001; TLR4-KO mice: 20.3 ± 3.3 for B2M group vs. 37.5 ± 9.4 for Veh group, *p* = 0.0010). TLR4 elimination also alleviated B2M-induced spatial memory dysfunction as indicated by more time in the target zone (22.01 ± 8.31 for B2M group of TLR4-KO mice vs. 13.28 ± 5.65 for WT mice, *p* = 0.0132).

### B2M-induced cognitive dysfunction was partially dependent on TLR4 activation

**Fig. 2:**
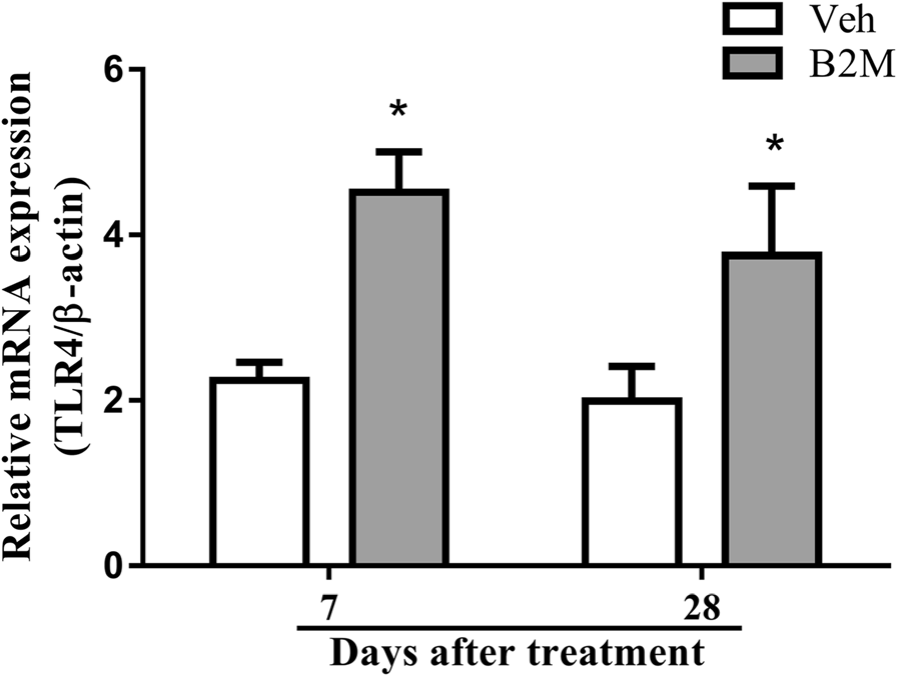
B2M-induced cognitive dysfunction was partially dependent on TLR4 activation. Expression of TLR4 mRNA in hippocampus of WT mice; β-actin was used as an endogenous reference gene. B2M induced a significantly higher expression of TLR4 mRNA in hippocampus (*n* = 6/subgroup). The data were analyzed using one-way ANOVAs with post-hoc pairwise comparisons. Values are presented as the means ± SD. Significant differences are expressed as follows: *p < 0.0001 vs. Veh group in WT mice. WT = wild type C57BL/6

To explore the role of TLR4 in the course of B2M-induced cognition impairment, we measured TLR4 mRNA expression in the hippocampus of the WT mice from Veh group and B2M group on day 7 and 28 after B2M treated. Here, we observed significantly higher levels of TLR4 mRNA expression at day 7 and 28 after B2M treated (day 7: 4.52 ± 0.49 for B2M group vs. 2.24 ± 0.22 for Veh group, *p* < 0.0001; day 28: 3.75 ± 0.84 for B2M group vs. 1.99 ± 0.42 for Veh group, *p* < 0.0001; Fig. 2).

### TLR4 elimination increases adult hippocampus neurogenesis following B2M treatment

**Fig. 3:**
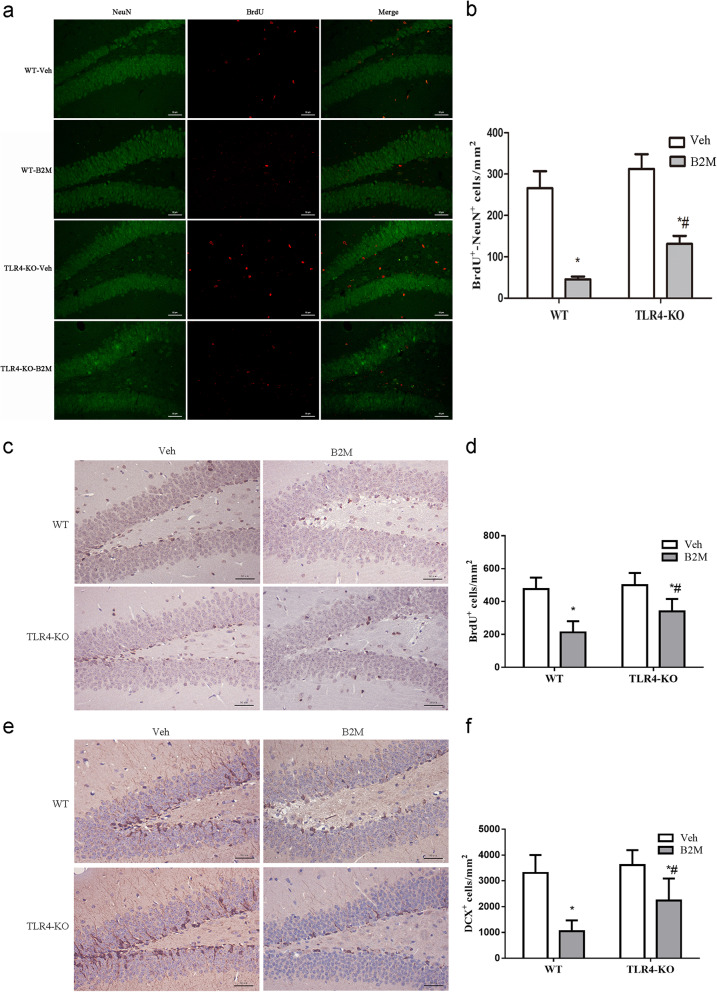
TLR4 elimination increases adult hippocampus neurogenesis following B2M treatment. The mature neurons (**a**), proliferating progenitor cell (**c**) and immature neurons (**e**) were assessed in the hippocampus DG region of all groups of mice (*n* = 4/subgroup). B2M decreased the number of BrdU/NeuN (**b**), BrdU (**d**) and DCX (**f**) positive cells in hippocampus DG, and which was reversed by TLR4 eliminations. The data were analysed using two-way ANOVAs used Tukey’s multiple comparisons test. Values are presented as the means ± SD. Significant differences are expressed as follows: *p < 0.05 compared B2M group vs. Veh group of WT and TLR4-KO mice, ^#^*p* < 0.05 compared B2M group of TLR4-KO mice vs. WT mice, according to the two-way ANOVA. Original magnification: × 100(**a**); × 200 (**c**, **e**). Scale bar = 50 μm. BrdU = 5-bromo-2′ deoxyuridien, DCX^+^ = Doublecortin positive cells, PSD-95 = postsynaptic density protein 95, WT = wild type C57BL/6, TLR4-KO = TLR4 knockout

To explore the cellular basis for the observed changes in learning and memory, we investigated the effects of B2M and TLR4 on the production of neurons in the dentate gyrus (DG) of hippocampus. A double BrdU/NeuN immunostaining was performed 22 day after BrdU administration. As show in Fig. 3a, b, the number of double BrdU/NeuN positive cells was decreased in B2M group compared with Veh group of WT and TLR4-KO mice (WT mice: 46.30 ± 13.65 for B2M group vs. 260.00 ± 80.75 for Veh group, *p* < 0.0001; TLR4-KO mice: 150.30 ± 62.49 for B2M group vs. 287.40 ± 84.82 for Veh group, *p* = 0.0006). TLR4 elimination caused an improvement in neurogenesis, indicated by increased BrdU/NeuN positive cells (150.30 ± 62.49 for B2M group of TLR4-KO mice vs. 46.30 ± 13.65 for WT mice, *p* < 0.0001).

To understand if the improvement hippocampus neurogenesis was the result of increased progenitor cell proliferation and immature neurons, the number of BrdU positive cells (BrdU^+^) and immature neurons (Doublecortin positive cells, DCX^+^) in hippocampus DG was examined at 7 day after B2M treated (Fig. 3c, e). BrdU^+^ and DCX^+^ cells were decreased in B2M group of WT and TLR4-KO mice compared with Veh group (BrdU^+^ cells: 212.3 ± 67.83 for B2M group vs. 476.0 ± 69.42 for Veh group of WT mice, *p* = 0.0016; 339.4 ± 76.20 for B2M group vs. 499.8 ± 74.08 for Veh group of TLR4-KO mice, *p* = 0.0234; DCX ^+^ cells: 1046.1 ± 421.92 for B2M group vs. 3306.5 ± 695.79 for Veh group of WT mice, *p* = 0.0014; 2237.9 ± 851.10 for B2M group vs. 3608.9 ± 582.13 for Veh group of TLR4 KO-mice, *p* = 0.0376; Fig. 3d, f). Meanwhile, TLR4 elimination also caused an increased in BrdU and DCX positive cells (BrdU^+^ cells: 339.4 ± 76.20 for B2M group of TLR4-KO mice vs. 212.3 ± 67.83 for WT mice, *p* = 0.0471; DCX^+^ cells: 2237.9 ± 851.10 for B2M group of TLR4-KO mice vs. 1046.1 ± 421.92 for WT mice, *p* = 0.0460; Fig. 3d, f).

### TLR4 elimination increased the level of synaptophysin (SYN) and PSD-95 following B2M treatment

**Fig. 4:**
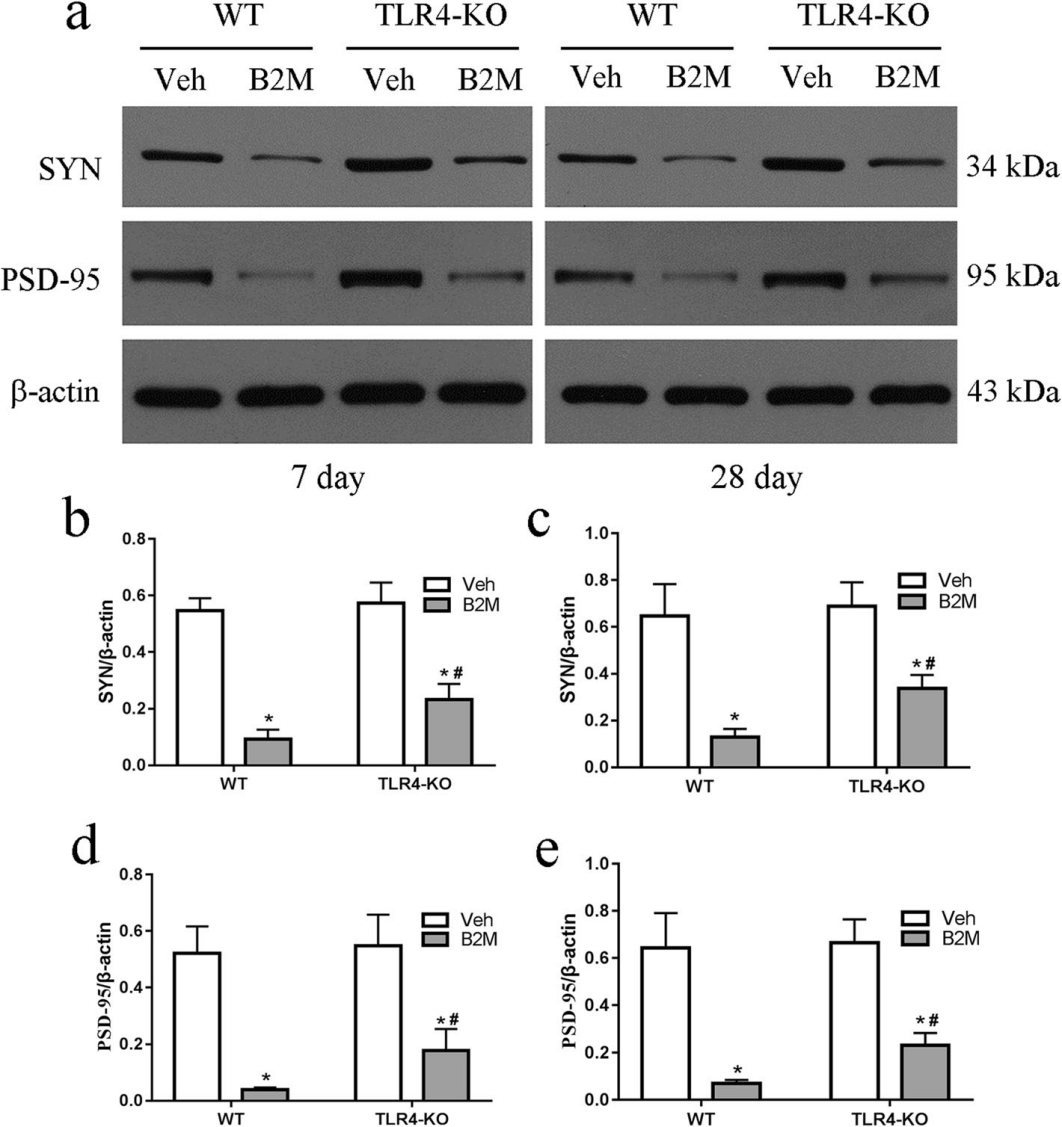
TLR4 elimination increased the level of SYN and PSD-95 following B2M treatment. The intensity of synaptophysin and PSD-95 in the hippocampus were determined at 7 and 28 days after B2M or vehicle treatment (*n* = 6/subgroup). The relative intensity in arbitrary units compared to β-actin (**a**). B2M treatment decreased the levels of synaptophysin and PSD-95 were at 7 (**b**, **d**) and 28 days (**c**, **e**), which was improved in TLR4-KO mice. The data were analyzed using two-way ANOVAs used Tukey’s multiple comparisons test. Values are presented as the means ± SD. Significant differences are expressed as follows: *p < 0.05 compared B2M group vs. C group in WT and TLR4-KO mice, according to the two-way ANOVA; ^#^*p* < 0.05 compared B2M group in TLR4-KO mice vs. WT mice, according to the two-way ANOVA. SYN = synaptophysin, WT = wild type C57BL/6, TLR4-KO = TLR4 knockout

Synaptophysin is a presynaptic protein, while PSD-95 is a scaffolding protein in the postsynaptic density, both of which play critical roles in synaptic plasticity and cognition [17]. To determine if the B2M and TLR4 are associated with altered synaptic integrity, we examined the protein levels of SYN and PSD-95 in hippocampus using Western blot (Fig. 4a). Original full western blot images were in supplementary Fig. 4a from the Additional file 1.

As shown in Fig. 4, the levels of SYN and PSD-95 were decreased in WT and TLR4-KO mice at 7 and 28 days after B2M treatment (day7: SYN: 0.09 ± 0.03 for B2M group vs.0.55 ± 0.04 for Veh group of WT mice, *p* < 0.0001; 0.23 ± 0.05 for B2M group vs. 0.57 ± 0.07 for Veh group of TLR4-KO mice, *p* < 0.0001; PSD-95: 0.04 ± 0.01 for B2M group vs. 0.52 ± 0.10 for Veh group of WT mice, *p* < 0.0001; 0.18 ± 0.08 for B2M group vs. 0.55 ± 0.11 for Veh group of TLR4-KO mice, *p* < 0.0001, Fig. 4b, d; day28: SYN: 0.12 ± 0.03 for B2M group vs.0.65 ± 0.14 for Veh group of WT mice, *p* < 0.0001; 0.34 ± 0.06 for B2M group vs. 0.69 ± 0.10 for C group of TLR4-KO mice, *p* < 0.0001; PSD-95: 0.07 ± 0.01 for B2M group vs. 0.64 ± 0.15 for Veh group of WT mice, *p* < 0.0001; 0.23 ± 0.05 for B2M group vs. 0.66 ± 0.10 for Veh group of TLR4-KO mice, *p* < 0.0001, Fig. 4c, e). Both the levels of SYN and PSD-95 are improved in TLR4-KO mice after B2M treatment (day7:SYN: 0.23 ± 0.06 for B2M group of TLR4-KO mice vs. 0.09 ± 0.03 for WT mice, *p* = 0.0011; PSD-95: 0.18 ± 0.08 for B2M group of TLR4-KO mice vs. 0.04 ± 0.01 for WT mice, *p* = 0.0384, Fig. 4b, d; day28: SYN: 0.34 ± 0.06 for B2M group of TLR4-KO mice vs. 0.12 ± 0.03 for WT mice, *p* = 0.0041; PSD-95: 0.23 ± 0.05 for B2M group of TLR4-KO mice vs. 0.07 ± 0.01 for WT mice, *p* = 0.0326, Fig. 4c, e).

In cultured primary neurons, we further investigated this function, the expressions of proteins of SYN and PSD-95 were detected using Western blot (Fig. 7a, d, e). After incubation, the protein expression levels of SYN and PSD-95 were significantly declined in B2M neurons, which were attenuated in TLR4-KO group (all *p* < 0.0001). Original full western blot images of SYN and PSD-95 were in supplementary Fig. 7 from the Additional file 1.

### TLR4 elimination decreased the expression of IL-1β, TNF-α following B2M treatment

**Fig. 5:**
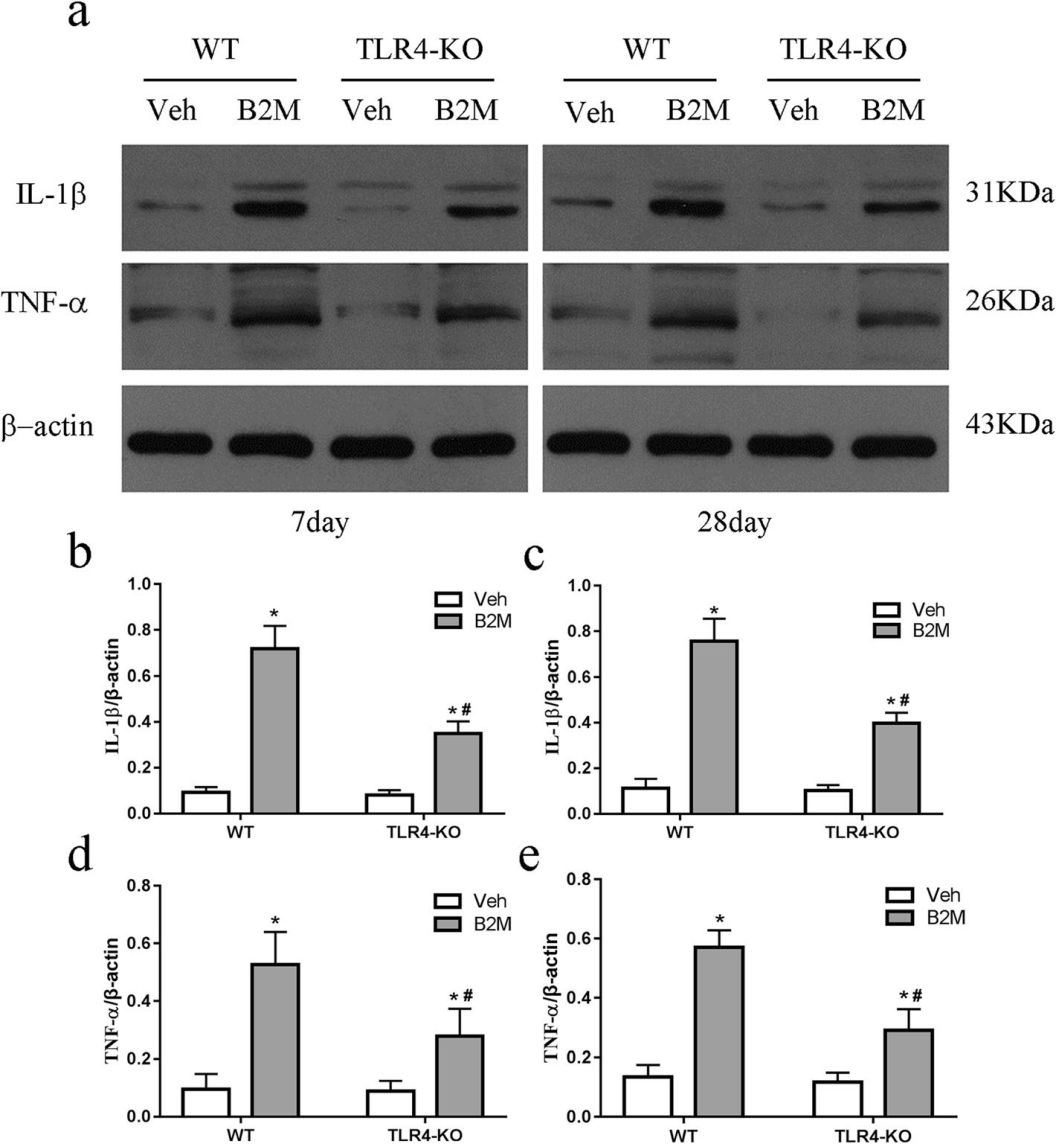
TLR4 elimination decreased the expression of IL-1β, TNF-α following B2M treatment. IL-1β and TNF-α protein expression in the hippocampus were determined at 7 and 28 days after B2M or vehicle treatment (*n* = 6/subgroup). The relative level in arbitrary units compared to β-actin (**a**). B2M treatment increased the levels of IL-1β and TNF-α were at 7 (**b**, **d**) and 28 days (**c**, **e**), which was reversed in TLR4-KO mice. The data were analyzed using two-way ANOVAs used Tukey’s multiple comparisons test. Values are presented as the means ± SD. Significant differences are expressed as follows: *p < 0.05 compared B2M group vs. Veh group in WT and TLR4-KO mice, according to the two-way ANOVA; ^#^*p* < 0.05 compared B2M group in TLR4-KO mice vs. WT mice, according to the two-way ANOVA. IL-1β = interleukin-1β, TNF-α = tumor necrosis factor-alpha, WT = wild type C57BL/6, TLR4-KO = TLR4 knockout

We measured the levels of the inflammatory cytokines IL-1β, TNF-α in the hippocampus to investigate the role of TLR4 on inflammatory response following B2M treated (Fig. 5) using Western Blot. At 7 and 28 day after B2M treated, the levels of IL-1β and TNF-α in the hippocampus significantly increased in the WT and TLR4-KO mice (day7: IL-1β: 0.72 ± 0.10 for B2M group vs. 0.09 ± 0.02 for Veh group of WT mice, *p*<0.0001; 0.34 ± 0.05 for B2M group vs. 0.08 ± 0.02 for Veh group of TLR4-KO mice, *p*<0.0001;TNF-α: 0.53 ± 0.11 for B2M group vs. 0.10 ± 0.05 for Veh group of WT mice, *p*<0.01; 0.28 ± 0.10 for B2M group vs. 0.09 ± 0.04 for Veh group of TLR4-KO mice, *p*<0.01; day28: IL-1β: 0.76 ± 0.10 for B2M group vs.0.11 ± 0.04 for Veh group of WT mice, *p*<0.0001; 0.40 ± 0.05 for B2M group vs. 0.10 ± 0.02 for Veh group of TLR4-KO mice, *p*<0.0001; TNF-α: 0.57 ± 0.06 for B2M group vs. 0.13 ± 0.04 for Veh group of WT mice, *p*<0.0001; 0.29 ± 0.07 for B2M group vs. 0.12 ± 0.03 for Veh group of TLR4-KO mice, *p*<0.0001), which was attenuated in TLR4-KO mice (day7: IL - 1β: 0.34 ± 0.05 for B2M group of TLR4-KO mice vs. 0.72 ± 0.10 for WT mice, *p*<0.0001; TNF-α: 0.28 ± 0.10 for B2M group of TLR4-KO mice vs. 0.53 ± 0.11 for WT mice, *p*<0.01; day28: IL-1β: 0.40 ± 0.05 for B2M group of TLR4-KO mice vs. 0.76 ± 0.10 for WT mice, *p*<0.0001; TNF-α: 0.27 ± 0.07 for B2M group of TLR4-KO mice vs. 0.57 ± 0.06 for WT mice, *p p*<0.0001). Original full western blot images were in supplementary Fig. 5a from the Additional file 1.

Cultured primary neurons showed almost the same trend, as shown in Fig. 7a~c, the level of IL-1β and TNF-α was increased when after B2M-treated, and these effects were reversed in TASK-KO mice (all *p* < 0.0001). Original full western blot images of IL-1β and TNF-α were in supplementary Fig. 7 from the Additional file 1.

### TLR4 elimination decreased the apoptototic neuronal death following B2M treatment

**Fig. 6:**
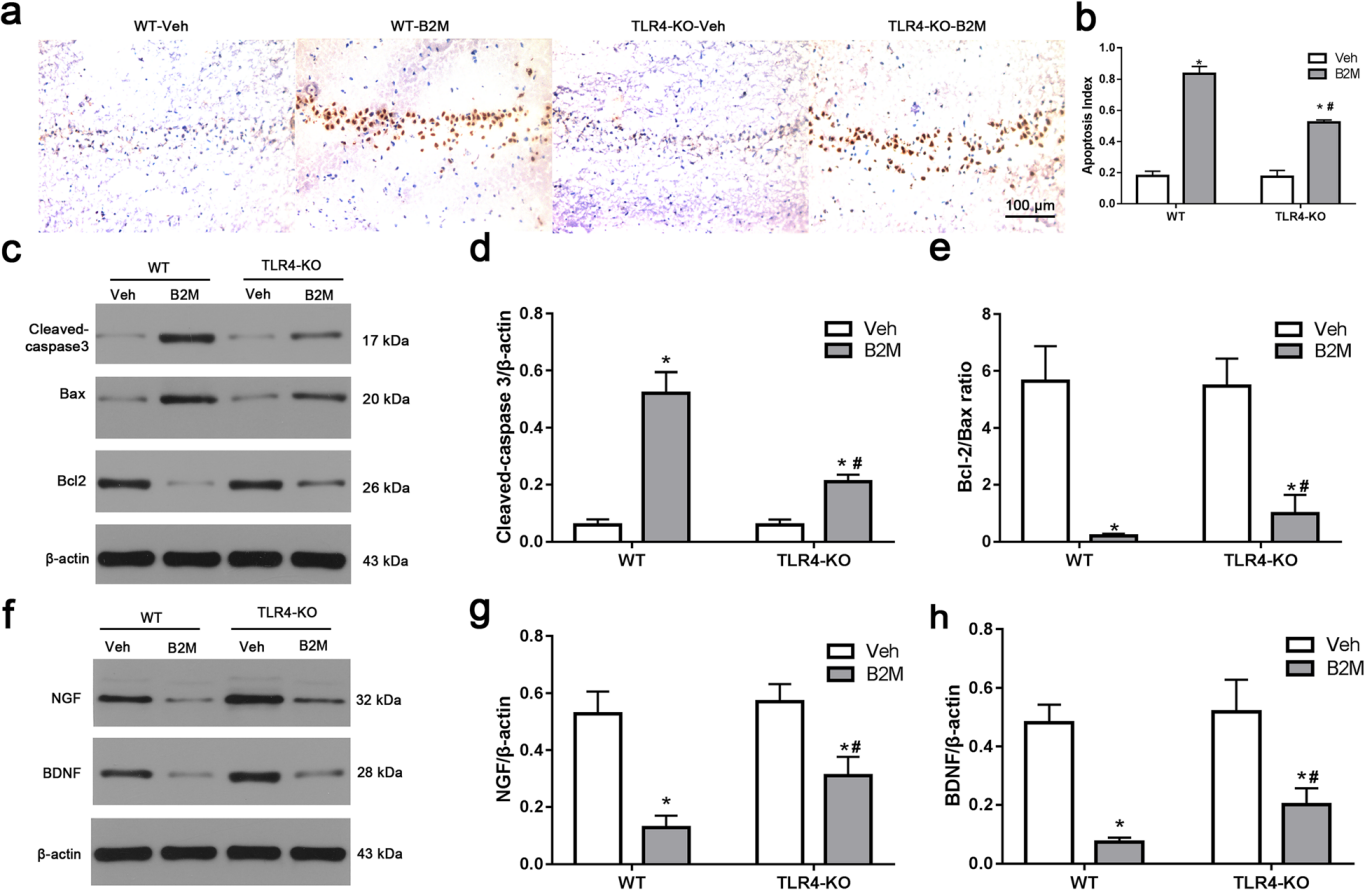
TLR4 elimination decreased the apoptototic neuronal death and increased the neurotrophic factors following B2M treatment. **a** Representative images of TUNEL-stained sections in hippocampal CA1 region of mice. Data were obtained from three independent animals per group, and the results of a typical result are exhibited. Scale bar = 100 μm. (*n* = 3/subgroup) (**b**) Quantitative analysis of the apoptosis index in hippocampal CA1 region. **c**-**e** Western blot analysis of apoptosis-related proteins cleaved-caspase-3, Bax and Bcl-2 in vivo experiment, β-actin was used as an internal control (*n* = 6/subgroup). **f**-**h** Western blot analysis of NGF and BDNF of hippocampus in vivo experiment, β-actin was used as an internal control (*n* = 6/subgroup). The data were analyzed using two-way ANOVAs used Tukey’s multiple comparisons test. Values are presented as the means ± SD. Significant differences are expressed as follows: *p < 0.05 compared B2M group vs. Veh group in WT and TLR4-KO mice, according to the two-way ANOVA; ^#^*p* < 0.05 compared B2M group in TLR4-KO mice vs. WT mice, according to the two-way ANOVA. WT = wild type C57BL/6, TLR4-KO = TLR4 knockout

We further performed TUNEL-staining to detect apoptotic neuronal death (Fig. 6a). Neurons with normal morphology were observed in hippocampus of Veh mice. However, TUNEL-positive neurons were increased in B2M-treated mice and TLR4-KO markedly decreased TUNEL-positive neurons number after B2M-treated. Statistic results showed that the apoptosis index of B2M-treated mice was significantly higher than that of Veh in WT mice (0.83 ± 0.05 vs. 0.18 ± 0.03, *p* < 0.0001), and this was partially diminished in TLR4-KO mice after B2M-treated (0.52 ± 0.02 vs. 0.83 ± 0.05, *p* < 0.0001 vs. B2M in WT) (Fig. 6b).

**Fig. 7:**
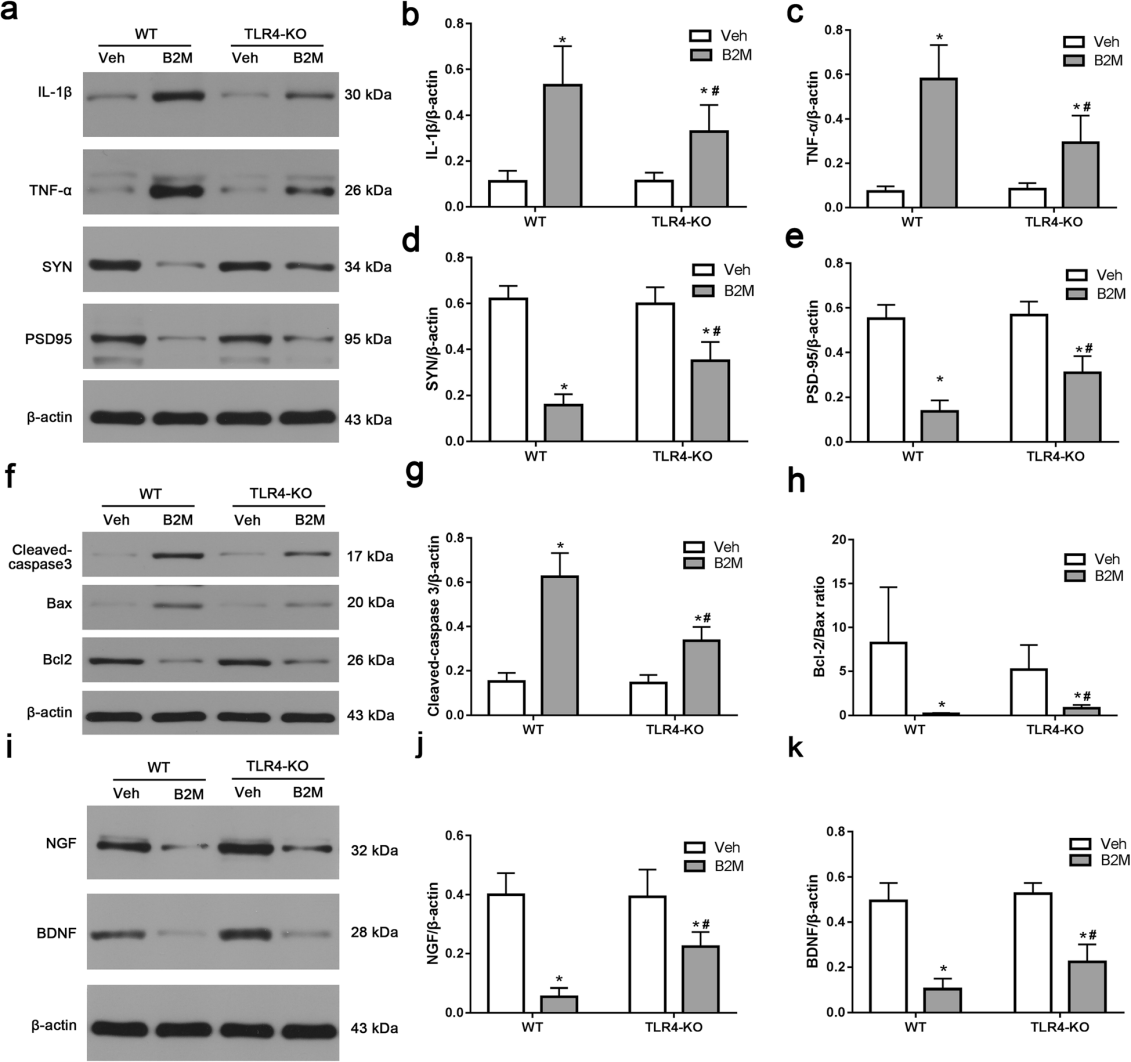
TLR4 elimination decreased the neuroinflammation, apoptosis, increased the level of synaptic proteins and neurotrophic factors following B2M treatment in cultured primary neurons. B2M treatment increased the expression of IL-1β, TNF-α, cleaved-caspase3, and decreased the level of SYN, PSD-95, NGF BDNF and Bcl-2/Bax ratio, which was attenuated in TLR4-KO neurons. **a**-**e** Western blot analysis of IL-1β, TNF-α, SYN and PSD-95 in cultured primary neurons, β-actin was used as an internal control(*n* = 6/subgroup). **f**-**h** Western blot analysis of apoptosis-related proteins cleaved-caspase-3, Bax and Bcl-2 in cultured primary neurons, β-actin was used as an internal control(*n* = 6/subgroup). **i**-**k** Western blot analysis of NGF and BDNF in cultured primary neurons, β-actin was used as an internal control(*n* = 6/subgroup). The data were analyzed using two-way ANOVAs used Tukey’s multiple comparisons test. Values are presented as the means ± SD. Significant differences are expressed as follows: *p < 0.05 compared B2M group vs. Veh group in WT and TLR4-KO mice, according to the two-way ANOVA; ^#^*p* < 0.05 compared B2M group in TLR4-KO mice vs. WT mice, according to the two-way ANOVA. WT = wild type C57BL/6

We also assessed whether apoptotic responses were related to TLR4 and B2M both in vivo (Fig. 6c-e) and in vitro (Fig. 7f-h). The Bcl-2/Bax ratio was decreased after B2M treatment in WT, whereas these effects were reversed in TASK-KO group (all *p* < 0.0001 vs. B2M in WT). Moreover, the activation of cleaved-caspase3 was significantly ameliorated in TLR4-KO group after B2M treatment. Original full western blot images of cleaved-caspase3, Bcl-2 and Bax in vivo and vitro were respectively in supplementary Fig. 6c and Fig 7 from the Additional file 1.

### TLR4 elimination increased the level of nerve growth factor (NGF) and brain-derived neurotrophic factor (BDNF) following B2M treatment

Next, we examined the protein levels of NGF and BDNF in hippocampus using Western blot.

As shown in Fig. 6f-h, the two-way ANOVA revealed a significant main effects for B2M (*p* < 0.001) and for genotype (*p* < 0.001) in the hippocampus, the levels of NGF and BDNF were decreased in WT mice at 7 days after B2M treatment (NGF: 0.129 ± 0.041 for B2M group vs.0.529 ± 0.078 for Veh group of WT mice, *p* < 0.0001; BDNF: 0.074 ± 0.015 for B2M group vs. 0.482 ± 0.061 for Veh group of WT mice, *p* < 0.0001), which the decrease was attenuated in TLR4-KO mice after B2M treatment (NGF: 0.310 ± 0.066 for B2M group of TLR4-KO mice vs. 0.129 ± 0.041 of WT mice, *p* = 0.0004; BDNF: 0.257 ± 0.019 for B2M group of TLR4-KO mice vs. 0.165 ± 0.019 for WT mice, *p* < 0.0001). Original full western blot images were in supplementary Fig. 6f from the Additional file 1.

To further explore the effect of B2M on cultured primary neurons, the expressions of proteins of NGF and BDNF were detected using Western blot (Fig. 7i-k). After incubation, the protein expression levels of NGF and BDNF were significantly declined in B2M group (all *p* < 0.0001), and TLR4-KO group showed a slight increase in the levels of NGF and BDNF (all *p* < 0.0001). Original full western blot images of NGF and BDNF were in supplementary Fig. 7 from the Additional file 1.

### TLR4 elimination inhibited the B2M-induced activation of TLR4/MyD88/NF-κB signaling pathway in hippocampal neurons

**Fig. 8:**
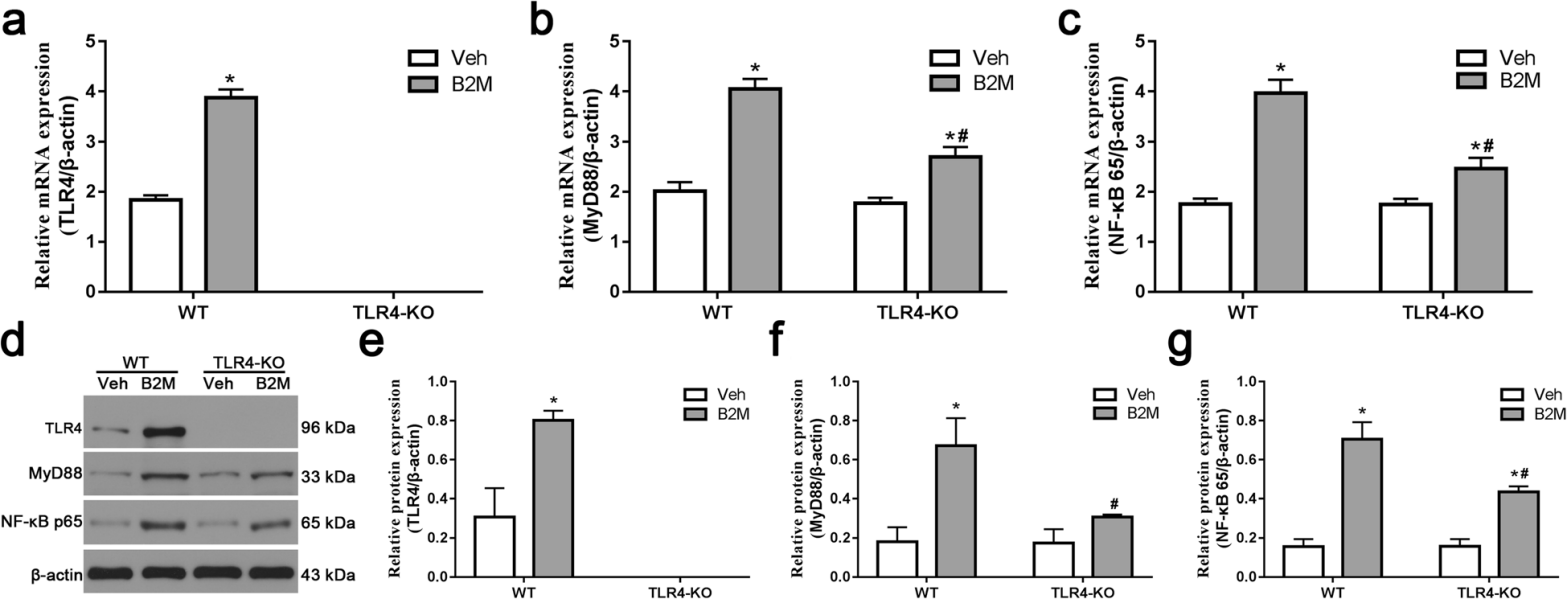
TLR4 elimination inhibited the B2M-induced activation of TLR4/MyD88/NF-κB signaling pathway in cultured primary neurons. TLR4 elimination inhibited the activation of TLR4/MyD88/NF-κB signaling pathway after B2M-treatment in cultured primary neurons. (**a**-**c**) Expression of TLR4, MyD88 and NF-κB mRNA in cultured primary neurons. (**d**-**g**) Western blot analysis of TLR4, MyD88 and NF-κB protein in cultured primary neurons, β-actin was used as an internal control (*n* = 3/subgroup). The data were analyzed using two-way ANOVAs used Tukey’s multiple comparisons test. Values are presented as the means ± SD. Significant differences are expressed as follows: *p < 0.05 compared B2M group vs. Veh group in WT and TLR4-KO mice, according to the two-way ANOVA; ^#^*p* < 0.05 compared B2M group in TLR4-KO mice vs. WT mice, according to the two-way ANOVA. WT = wild type C57BL/6

To further analyze the regulatory mechanism of B2M-induced age-related cognitive decline, the expressions of the key nodes (TLR4, MyD88 and NF-κB) in TLR4 signaling pathway were detected (Fig. 8). After incubation with B2M, the expression levels of TLR4, MyD88 and NF-κB were significantly elevated compared with those in the Veh group (all *p* < 0.0001), which were inhibited in TLR4-KO group after B2M-treated (*p* < 0.0001). These results suggest that B2M activates TLR4 signaling pathway and also imply that the regulation of B2M-induced age-related cognitive decline are probably mediated through TLR4 signaling pathway. Original full western blot images were in supplementary Fig. 8 from the Additional file 1.

## Discussion

It should be noted that age-related cognition decline may increase the probability of various age-associated diseases and neurodegenerative dysfunctions, such as Parkinson’s disease, mild cognitive impairment, AD, and other dementias [18]. However, the mechanisms of these age-associated declines are unknown. An analysis of the factors of aged-related cognitive impairment may improve the clinical patients of age-associated declines.

We have analyzed cognitive function by MWM [19], consistent with previous studies [8], we found here a significant dysfunction of spatial learning and memory after B2M treatment, which verified that increased systemic levels of B2M have participated in cognitive function in young mice. In addition, we have demonstrated that transgenic TLR4 elimination rescues this B2M-treated phenotype. The dependence of the negative association between B2M-induced aging and cognitive performance on the presence of TLR4 observed in this study suggests that TLR4 may be important and could function as a regulator of B2M-induced age-related declines. Studies with animal models have suggested that age-related cognitive dysfunction points to hippocampus defects [20], this study was designed to observe structural changes in the hippocampal 7 day and 22 day after treatment. The microscopic results demonstrated that hippocampal injury is attenuated in TLR4 KO mice than in WT mice. These data confirm TLR4 contributed to B2M-induced age-related cognition dysfunction.

Additionally, this improved B2M-induced age-related cognitive behavior in TLR4-KO animals, whether owing to a neuroinflammation and apoptosis mechanism, requires further proceeding. Previous study shown B2M could be a marker to detect the TLR4 [21], but how they related has not been fully confirmed. In the present study, we found that B2M increased TLR4 mRNA expression in the hippocampus of WT mice for the first time, demonstrating TLR4 may be an important pathway for B2M to be a participant in age-related cognitive decline. B2M plays a critical role in inflammation and apoptosis [22, 23]. Moreover, TLR4 mediates an inflammatory and apoptosis response, when TLR4 endocytosis impaired, the production of TLR-triggered proinflammatory cytokines and neuronal apoptosis were attenuated [9]. In this study, we found that the hippocampal levels of proinflammatory cytokines IL-1β and TNF-α, the number of TUNEL-positive cells and Caspase-3, Bax protein were significantly higher after B2M treated, while the Bcl-2 was decreased, and transgenic TLR4 elimination showed an opposite condition. These results suggest that the neuroinflammation and apoptosis activated by B2M-treated was through TLR4. Persistent inflammation in the immune system can impose injurious impacts to the CNS, and neuroinflammation might be a consequence of this to accelerate the progressive deterioration of age-related cognitive decline [24]. Moreover, Apoptosis has been proposed to explain the cell loss observed in numerous neurological disorders, including age-related cognitive decline [25]. In humans, hypomethylation of IL-1β is strongly associated with aging and upregulation of IL-1β contributes to age-related cognitive decline [26]. Furthermore, elevated systemic TNF-α produces acute cognitive dysfunction and inhibition TNF-α trafficking prevents cognitive decline in an Alzheimer’s disease mouse model [27]. Our previous study also demonstrated that MV-induced neuroinflammation affected postoperative memory dysfunction and TLR4 knockout ameliorates neuroinflammation. It has also been shown that aging promotes neuronal apoptosis (Pourmemar et al., 2017) possibly, through an increase in Caspase-3 protein expression and the increase of Bax/Bcl-2 ratio [25]. Additionally, mice lacking TLR4 show reduced neuronal apoptosis and decreased pathology in the brain [9]. These demonstrated that the attenuated age-related cognitive decline after B2M-treated found in TLR4 KO mice are partly due to reductions in neuroinflammation and apoptosis.

On a mechanistic ground, the aged-related impairment of learning and memory has been demonstrated to involved modulation of hippocampal neurogenesis [28]. In previous study, the accumulation of B2M in the hippocampus also could induced the decrease of neural progenitor cells and newly born neurons [8]. Adult hippocampal neurogenesis is a process that originates from precursor cells and results in new granule cell neurons. As the dividing cells, neural stem cells could be labeled with the proliferation marker BrdU, which persists in daughter cells [29]. And toward the final stage of neuronal differentiation, the newborns cells begin to express proteins typically present in mature neurons such as the nuclear neuronal marker NeuN [29]. DCX, a marker of immature neuron, whose expression associated with migration of neuroblasts, at which the maturing granule cells become postmitotic [30]. In order to know whether the decrease net neurogenesis may result from the decrease number of neural stem cells and immature new neurons, we quantified the BrdU-labeled cells and DCX- labeled cells. As expected, the numbers of these BrdU-positive cells and DCX-positive cells were significantly reduced after B2M treatment. In addition, all of changes were less severe in TLR4 KO mice. A correlation has been shown between aging-related cognitive decline in healthy older adults and elevated plasma levels of IL-6 and Bax/Bcl ratio [31, 32]. Moreover, IL-1β, TNF-α production and an increase in the apoptosis can reduce adult hippocampal neurogenesis and produce the age-related cognitive decline [33, 34]. When TLR4 was activated by B2M in our study, the production of TLR-triggered proinflammatory cytokines and apoptosis were increased to activate microglia, which can reduce neurogenesis by suppressing neuronal stem cell proliferation, increasing apoptosis of neuronal progenitor cells, and decreasing survival of newly developing neurons and their integration into existing neuronal circuits, all of these is linked to age-related cognitive decline [35, 36]. These results suggested that neuroinflammation and apoptosis activated by TLR4 was implicated in age-related cognitive decline after B2M-treated through the hippocampal neurogenesis.

In humans, aging-related cognitive dysfunction may be caused by disturbances of synaptic integrity in the hippocampus [17]. B2M participated in synaptic function of the mammalian nervous system [37]. Researches indicate that TNF and IL-1β modulate synapses during neuroinflammation, and apoptosis is necessary for synaptic processes and underlying cognitive function [38]. When brain levels of neuroinflammations (IL1-β and TNF) and apoptosis significantly increased, whose essential activity is to maintain the proper synaptic function, they would start to damage the synaptic transmission [39]. In previous studies, as a postsynaptic protein and glutamate receptor, PSD-95 was significantly decreased in the hippocampus at 30 months of age, when the spatial cognitive deficits were exacerbated [17]. Synaptophysin is a presynaptic protein that also plays a critical role in synaptic function and related cognition. Reduced expression of SYP and PSD95 were marked feature in age-related cognitive decline [40], the synaptic protein loss may contribute to poor abilities of neurons to deal with the synaptic signaling transmission between the presynaptic and the postsynaptic membranes and cause synaptic dysfunction [41], which could result in the age-related cognitive decline in our study. Moreover, the decrease of pro-inflammatory cytokines expression and microglia activation improved the level of synaptophysin, which could upregulate the cognitive deficit in AD. Notably, the decrease of neuroinflammation and apoptosis resulted from elimination of TLR4 receptors was along with synaptic as well as cognitive decline [42]. In this study, we found that synaptic injury after B2M treated was attenuated in TLR4-KO mice, as the expressions of synaptophysin and PSD-95 are higher, which supported that TLR4 inhibition decreased B2M-induced neuroinflammation and apoptosis, and resulted in synaptic alteration in age-related cognitive decline after B2M-treated.

B2M is responsible for systemic amyloidosis, which can dysregulate the levels of BDNF and NGF [43]. In our study, the levels of BDNF and NGF were significantly reduced after B2M treatment, which were reversed in TLR4 KO mice. BDNF and NGF are crucial to synaptic plasticity, neuronal growth and neuronal survival [43]. Moreover, they were found to have decreased with aging and might contribute to an age-related decline in cognitive function [44]. In the aging, increased neuroinflammation induced by TLR4 would be associated with neurotrophic loss changes [45], and the increase level of BDNF, NGF in TLR4-KO group of our study would alleviate neuroinflammation [46] and apoptosis [47, 48] in the hippocampus which can ameliorate the reduced neuronal growth and neuronal survival by increasing the protection from damage [49] and result in the reduced cognitive impairment [18].

Our cell results further demonstrated that deletion of TLR4 could induce more potent neuroprotective effect and tend to significantly increase synaptic function, decrease neuroinflammtion and the apoptototic neuronal death as well as improve the level of BDNF and NGF after B2M-treatment. This potent neuroprotective effects may partly through the inhibition of neuroflammtion and apoptosis via the TLR4/MyD88/NF-κB signaling pathway.

Some limitations existed in our study. First, initiation of B2M treatment may represent a limitation because the half time of drugs, and measurement indicators levels were not fully rescued in TLR4 KO mice, other factors affected the B2M-induced cognitive decline should be researched in the future. Second, cognitive decline model cannot completely replicate the clinical age-related cognitive decline. Confusing elements have not be excluded, so further researches in which definite pathways participated are necessary to certificate our findings. Third, we did not observe the damage effects of TLR4 on B2M-induced age-related cognitive decline using agonist and we didn’t verify our research through systematical injection. Therefore, this condition should be the focus of future investigations. Finally, only hippocampus was investigated, other brain areas were required further measurement.

In this study, we found a pronounced increase of TLR4 expression in B2M-treated mice, which confirming a possible correlation with B2M-induced cognitive impairment. Conversely, TLR4 KO can counteract the B2M-induced cognitive decline due to neuroinflammation and apoptosis via a modulation of hippocmapal neurogenesis, synaptic plasticity through TLR4/MyD88/NF-κB signaling pathway. This may provide an importantly neuropotective mechanism for the improved age-related cognitive decline and also help devising new therapeutic to improve learning and memory function in the elderly individuals.

## Materials and methods

### Animal studies

All animal protocols were reviewed and approved by the Animal Ethics Committee of the Zhongnan Hospital and Research Center in Hubei, China, and all animal experiments complied with the ARRIVE guidelines and was carried out in accordance with the National Institutes of Health guide for the care and use of Laboratory animals (NIH Publications No. 8023, revised 1978). All efforts were made to minimize animal suffering and reduce the number of animals used.

### Animals

8–12 weeks male WT (C57BL/6) mice and TLR4-KO (C57BL/10ScNNju, J000192) mice weighing between 20 and 25 g were purchased respectively from Model Animal Research Center of Nanjing University in Jiangsu, China. As reported, TLR4 knockout mice are viable with no obvious spatial learning deficits in morris water maze paradigm (Uric Acid Induces Cognitive Dysfunction through Hippocampal Inflammation in Rodents and Humans). Both the WT and TLR4-KO mice were housed in groups of 3–5 each per cage, and in a light-controlled room (12 h light and 12 h dark cycle) at a temperature of 24 ± 1 °C and a humidity of 55% ± 5% with free access to food and water. The animals were allowed to adapt to new environment for at least 7 d before being used in experiments.

B2M (Sigma, St. Louis, MO, USA) was injected to young (8–12 weeks) mice by bilateral stereotaxic (Techman, DW-2000, Chengdu, China). WT and TLR4-KO mice were respective randomly divided into two groups: (1) vehicle group (Veh), which received vehicle (PBS, 0.5 μl) (Aspen, AS1025, Wuhan, China) in the bilateral DG of the dorsal hippocampus; (2) B2M-treated group(B2M), which received B2M (0.5 μl, 0.1 μg/μl) [8] in the bilateral DG of the dorsal hippocampus. B2M was freshly dissolved in PBS.

### Primary hippocampal neuron culture

Hippocampal neurons were prepared from postnatal day 1 of C57/BL and TLR4-KO mice under sterile conditions as reported by Brewer et al. [50] with further modifications. After mice were sacrificed, brain was removed and meninges were discarded, then hippocampus was separated from the cortex. Hippocampal tissue was cut in small pieces, transferred to papain solution (20 mg/ml), and incubated at 37 °C for 30 min with occasional gentle shaking. After 15 min, DNase I (50 μg/ml final concentration) was added. Tissue pieces were washed with Neurobasal medium and cell suspension was obtained using a fire-polished pipette in Neurobasal addemented and fetal bovine serum was used to terminate digestion. Cells were centrifuged at 160 g for 5 min and pellet was suspended in Neurobasal medium. Hippocampal cells were plated onto cell culture dish under a humified 37 °C incubator for 4 h, cultured in the same medium supplemented with 2 mM L-glutamine, 1 μg/ml gentamicin, 2% B27, and 10% FBS. Cells were cultured for 8 days in vitro for mature neurons in previous studies [51].

Carrier free forms of human recombinant B2M (Vendor) were dissolved in PBS and added to 3 ml cell culture medium (1 μg/ml) following cell plating. We changed half of the medium with 1.5 ml cell culture medium contained 1μg/ml B2M every other day, the B2M stayed in the cell cultures for 48 h each time till the end of the 8th day and the total dosage of B2M was 7.5 μg. Neurons were respective randomly divided into two groups: (1) vehicle group (Veh), which exposed to vehicle (PBS); (2) B2M-treated group(B2M), which exposed to soluble B2M [8].

### Drug delivery and BrdU labeling

For the B2M or vehicle treatment, all of the WT and TLR4-KO mice were anesthetized with 10% chloralic hydras (375 mg/kg, ip, Aspen, Wuhan, China) and placed in stereotaxic frame. B2M or vehicle was injected bilaterally into the DG of the dorsal hippocampus using the following coordinates: AP (anterior to posterior): − 2.0 mm, ML (media to lateral): ±1.5 mm, DV (dorsal to ventral): − 2.60 mm. To limit reflux along the injection track, the infusion needle was left in position for 8 min, slowly pulled out halfway and kept in position for an additional 2 min.

To label newborn cells, all mice received 5-bromo-2′ deoxyuridien (BrdU, 100 mg/kg, Boster, Wuhan, China) intraperitoneally once daily during the first six days after B2M or vehicle treatment. BrdU-labeled cells in the hippocampus were examined at 24 h or 22 days after the last BrdU injection.

### Morris water maze test

Morris water maze (MWM) was conducted 24 day after the B2M treatment as previously described [52]. The circular water maze pool (120 cm in diameter, 50 cm deep) was located in a dimly lit room. The water temperature was maintained at 26 ± 1 °C by a heating pad located beneath the pool. An escape platform (10 cm in diameter) was submerged about 1 cm below the water surface, in a fixed position in one of the quadrants (target zone quadrant). The pool was surrounded by curtains with distinct cues hung on them. Briefly, 10 mice of each group underwent the test at day 24 after B2M or vehicle treatment, including place navigation test for 4 days and spatial probe test for the last day. On each of four consecutive training days, mice were given four training of swimming with four different starting points, the middle of each of the four quadrants. The trail was complete once the mice climbed up the platform. If the mice were not able to find the platform within 60 s, the experimenter guided the mice onto the platform, and the time spent be recorded as 60 s. All of the mice were allowed to stay on the platform for 15 s. Time spent to find the platform (latency) during the training days were used to evaluate spatial learning and memory. At day 28 (spatial probe test) the platform was removed and the mice was allowed to search for one minute in the pool. Time travelled in the target zone and times across platform on the probe trail were used as an index of reference memory. Behaviors date in the training and the probe trail were acquired and analyzed using an automated tracking system (MicroPublisher, Panab company, Spain).

### Q-PCR for TLR4 mRNA

At 7 or 28 day after B2M or vehicle treatment, 6 mice in each group of WT mice were anesthetized with chloralic hydras and the hippocampus tissues were rapidly collected and stored at − 80 °C for processing. In vitra expereiment, according to the TRIfast standard procedure (EuroClone), Total RNA was obtained from each single cell population of *n* = 5 eyes/E-Reeler,, and resuspended in 10 μL fresh available RNAse-free water. After homogenization, total RNA was extracted using TRIzol reagent (Invitrogen™, USA). The cDNA was synthesized using PrimeScript™RT reagent kit (TaKaRa, RR047A, Dalian, China). For quantitative real-time PCR reaction, the SYBR Premix Ex Taq™ reagent kit (TaKaRa, RR420A, Dalian, China) was used in StepOne™ Real-Time PCR detection system (Life Technologies, USA). The PCR condition was pre-denaturation at 95 °C for 60 s, followed by 40 cycles of nannealing reaction at 95 °C for 15 s, and extension 60 °C for 20 s. β-actin was used as endogenous reference gene for normalizing the quantities of TLR4 gene expression in animals, and TLR4, MyD 88 and NF-κB 65 gene expression in cell.

The sequences of the primers used in this this study were as follows:

TLR4 forward: 5′-ACACTTTATTCAGAGCCGTTGGT-3′;

reverse: 5′-CAGGTCCAAGTTGCCGTTTC-3′.

MyD 88 forward: 5′-GGCATCTGCATATGTGTGTT-3′;

reverse: 5′- CCCAGGCTGACCTTAAACTA-3′.

NF-κB 65 forward: 5′- ACGACATTGAGGTTCGGTTC-3′;

reverse: 5′- ATCTTGTGATAGGGCGGTGT-3′.

β-actin forward: 5′-CTGAGAGGGAAATCGTGCGT-3′;

reverse: 5′-CCACAGGATTCCATACCCAAGA-3′.

### Immunostaining and image analysis

Four mice in each group were humanely sacrificed at 24 h or 22 days after the BrdU injection. Mice were anesthetized with chloralic hydras and perfused transcardially after a thoracotomy with 100 ml of ice-cold saline, followed by 4% paraformaldehyde (Aspen, Wuhan, China) at 4 °C. The fixed brain was rapidly removed, postfixed in 4% paraformaldehyde, embedded, and sectioned for immuostaining. 4 mice per group and 15 sections per mouse were analyzed, during section procedure, we choose every sixth sections mounting on slide for further analysis. For staining of BrdU-positive cells, sections were incubated with primary antibody (rabbit anti-BrdU, 1:300, Abcam, UK) and then biotin-conjugated secondary antibody (goat anti-rabbit IgG, 1:200, Aspen, USA). In order to estimate the total numbers of immature neurons, sections were incubated with primary antibody (rabbit anti-DCX, 1:200, Abcam, UK) and then biotin-conjugated secondary antibody (goat anti-rabbit IgG, 1:200, Aspen, USA). For BrdU-NeuN co-labeling, sections were multi-stained by incubation with primary antibodies (rabbit anti-BrdU at 1:100, or mouse anti-NeuN at 1:100, Abcam, UK) and then secondary antibodies (goat anti- rabbit IgG at 1:50, goat anti-mouse IgG at 1:50, Aspen, USA). Images were acquired using a fluorescence microscope (Olympus, Tokyo, Japan) equipped with an imaging system.

### Western blot analysis

The total protein samples from the hippocampal tissues and hippocampal neurons were homogenized using RIPA lysis buffer (150 mM NaCl, 1 mM EDTA, 50 mM Tris, 1% Triton, 0.1% sodium dodecyl sulfate, and 0.5% deoxycholate) containing protease and phosphatase inhibitors. The lysates were collected and then centrifuged at 13000 g at 4 °C for 5 min. The resulting supernatants were collected, and the protein concentrations were determined using a bicinchoninic acid protein assay (Apsen, USA). Sample protein (40 μg) were mixed with equal volumes of 5 × sodium dodecyl sulfate protein gel loading solution (Aspen, USA), boiled for 5 min. Aliquots of each sample (10 μl containing 40 μg protein) were loaded on 9–12% polyacrylamide gels and separated by electrophoresis for 90 min at 120 V. Separated proteins were transferred to polyvinylidene fluoride membrane (Millipore, USA) for 1.5 h at 300 mA. Nonspecific binding was blocked for 1 h at 37 °C with 5% nonfat dry milk in Tris-buffered saline Tween (TBST, 20 mM Tris, 150 mM NaCL, 0.1% Tween, pH 7.5). Membranes were incubated overnight at 4 °C with the following antibodies: rabbit anti-NGF (1:1000; Abcam, UK), rabbit anti-BDNF (1:1000; Abcam, UK), rabbit anti-synaptophysin (1:3000, Abcam, UK), rabbit anti-PSD-95 (1:1500, Abcam, UK), anti-IL-1β (1:500, Bioss, Beijing, China) and rabbit anti-TNF-α (1:1000, Abcam, UK), rabbit anti-Bcl-2 (1:1000; 2876, Cell Signaling Technology, USA), rabbit anti-Bax (1:1000; 2772, Cell Signaling Technology, USA), rabbit anti-cleaved-Caspase-3 (1:1000; 9661, Cell Signaling Technology, USA), rabbit Anti-MyD88(1:1000; Abcam, UK), rabbit anti-NF-κB p65 (1:1000; Abcam, UK). The membranes were washed 3 times with TBST at 37 °C for 15 min and then incubated with secondary antibodies (anti-rabbit IgG at 1:10000, Aspen, USA). Blots were developed using the enhanced chemiluminescence technique. Band densities were quantified using AlphaEaseFC software (Alpha Innotech, USA). β-actin (1:10000, Tdy Biotech, Beijing, China) was blotted on the same membrance as a loading control. The quantified values are expressed as a percentage to β-actin intensity.

### Terminal deoxynucleotidyl transferase-mediated dUTP-biotin nick end labeling (TUNEL) staining

At 7 day after B2M or vehicle treatment, mice (*n* = 3 per group) were deeply anesthetized and cardiac perfused with pre-cold heparinized-normal saline and 4% paraformaldehyde. After postfixed in the same fixative solution, brain samples were dehydrated, and then cryosectioned at a thickness of 20 μm. Every sixth sections through the longetutive hippocampus were selected for further study. Commercial InSitu Cell Death Detection Kit (Roche Diagnostics, Indianapolis, IN, USA) was employed to detect neuronal apoptosis according to the manufacturer’s instructions [53]. For apoptotic neuronal quantification,, an experienced examiner who was blinded to experimental design was invited to observe the sections under light microscopy (× 100 magnification), four microscope vision fields were visualized, total number of positive cells was counted using Image Pro Plus software. The neuronal apoptotic index was calculated and expressed as a percentage of TUNEL-positive neurons versus total neurons.

### Statistical analysis

Statistical analysis was performed using the SPSS 21.0 statistical software (SPSS Inc., USA). The data from Q-PCR in WT mice were analyzed using one-way ANOVAs with post-hoc pairwise comparisons, the data from Bcl-2/Bax ratio were analyzed using t tests. The other data were analyzed using two-way ANOVAs used Tukey’s multiple comparisons test. The results were expressed as mean ± standard deviation. *P* value less than 0.05 was considered to be statistically significant.

### Supplementary information

**Additional file 1:** This document contains additional information on the original full western blot images from all the samples.

## Data Availability

The data supporting the findings of this study are included within the article.

## Abbreviations

AD: Alzheimer’s disease
B2M: β2-microglobulin
BDNF: Brain-derived neurotrophic factor
BrdU: 5-bromo-2′ deoxyuridien
CNS: Central nervous system
DCX^+^: Doublecortin positive cells
DG: Dentate gyrus
IL-1: Interleukin-1
MWM: Morris water maze
NGF: Nerve growth factor
PSD-95: Postsynaptic density protein 95
SYN: Synaptophysin
TLR4: Toll-like receptor 4
TNF-α: Tumor necrosis factor-alpha
