# Correction to: Toll-like receptor 4 deficiency ameliorates β2-microglobulin induced age-related cognition decline due to neuroinflammation in mice

**DOI:** 10.1186/s13041-024-01090-w

**Published:** 2024-04-23

**Authors:** Qi Zhong, Yufeng Zou, Hongchao Liu, Ting Chen, Feng Zheng, Yifei Huang, Chang Chen, Zongze Zhang

**Affiliations:** 1 Department of Anesthesiology, Zhongnan Hospital, Wuhan University, East Lake Road, 430071 Wuhan, Hubei China; 2 Department of Anesthesiology, Maternal and Child Hospital of Hubei Province, Wuluo Road, 430071 Wuhan, Hubei China

**Correction to**: *Mol Brain***13**, 20 (2020).

10.1186/s13041-020-0559-8

Following publication of the original article [1], the authors identified an error in Fig. 3a. After carefully reviewing the original figures and data in four groups of Fig. 3a (NeuN and Brdu), the authors found that the Fig. 3a were presented erroneously and some pictures were not the final version. The authors apologize for any confusion this may have caused and have corrected these errors.

(1) In WT-Veh group, the authors apologize for the error in the inappropriate presentation of the BrdU picture, which was not the final version. The authors have now replaced the representative images of BrdU expression and merge in dentate gyrus (DG) of the hippocampus (middle and right panel) to the correct version.

(2) The quality of NeuN picture selected initially in TLR4-KO-B2M group was poor, so the authors have eliminated them and did not incorporated the pictures and data in the group. Then the authors have amended the three pictures (NeuN, Brdu and Merge) in the final version of TLR4-KO-B2M group. The authors regret that an incorrect version was published mistakenly due to their carelessness, now they have rectified this mistake and replaced the pictures to the correct ones.

The authors confirm that the overall conclusions reported in the paper remain unaffected and valid.

The incorrect and correct Fig. 3 are indicated hereafter.

The incorrect Fig. 3(a):


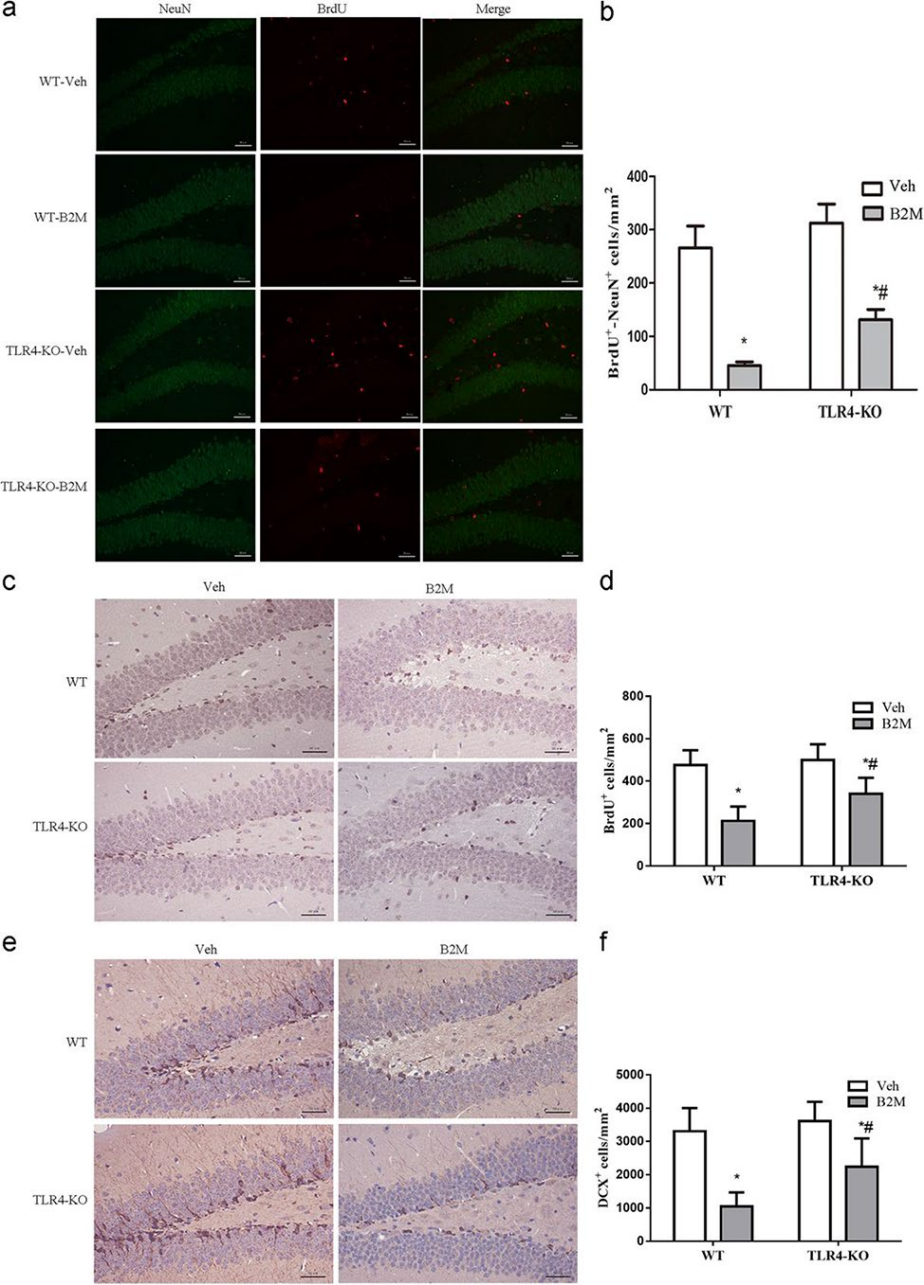


The correct Fig. 3a:

**Fig. 3:**
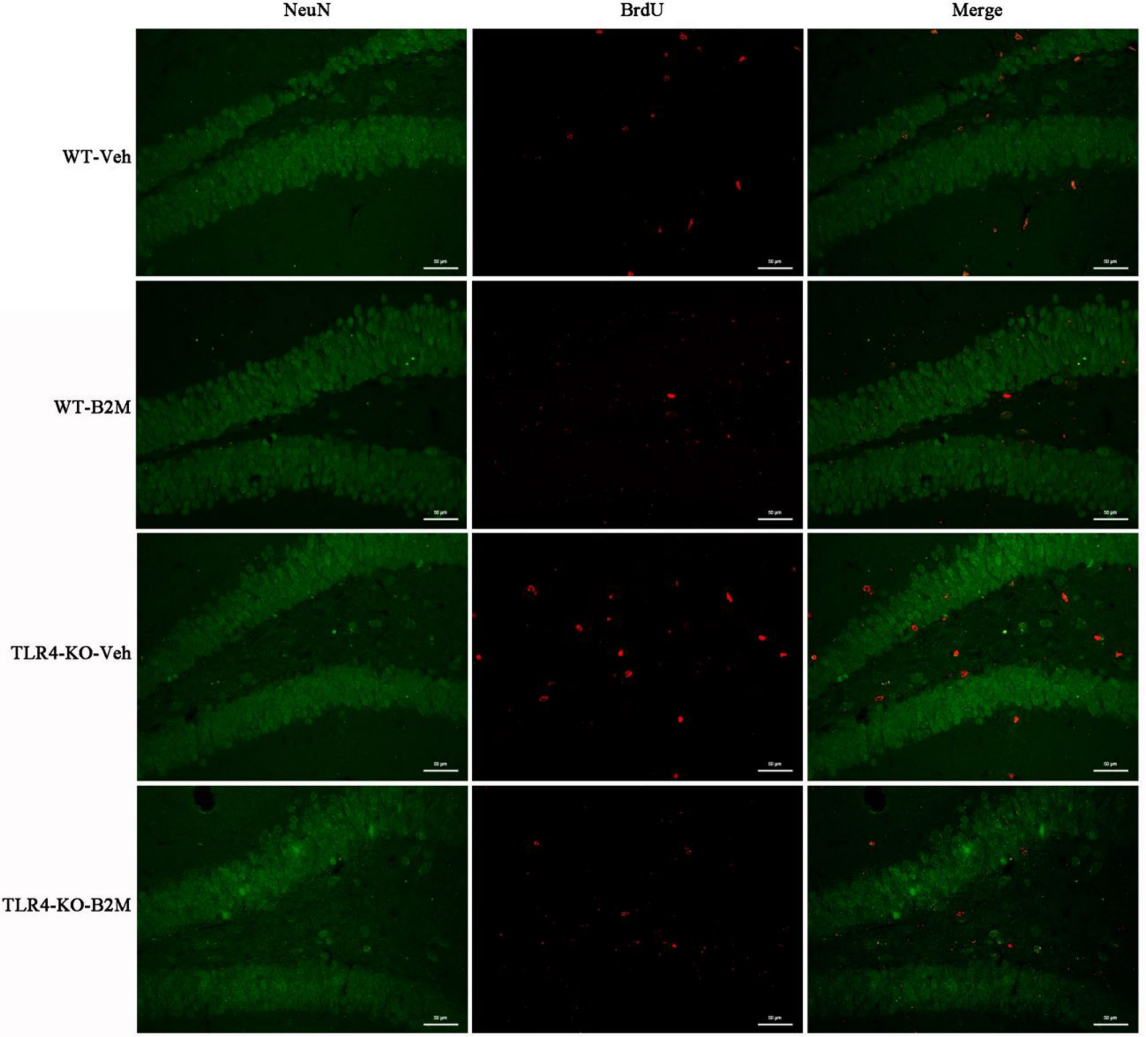
(**a**) has been updated above and the original article [1] has been corrected
