# Supplementary material for: Toll-like receptor 4 deficiency ameliorates β2-microglobulin induced age-related cognition decline due to neuroinflammation in mice
Source: Mol Brain. 2020 Feb 14;13:20. doi: 10.1186/s13041-020-0559-8 (PMC7023753; doi:10.1186/s13041-020-0559-8)

**SUPPLEMENTARY INFORMATION**

This document contains additional information on the original full western blot images from all the samples.

**Supplementary Fig 4a, 5a: original full western blot images**


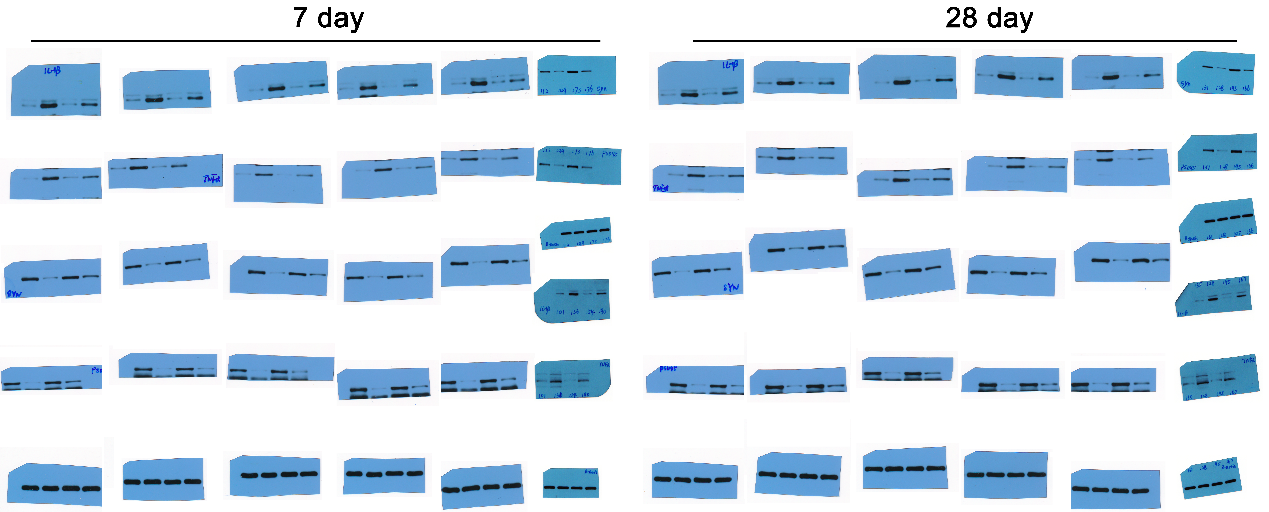


**Supplementary Fig 6c, 6f: original full western blot images**

**
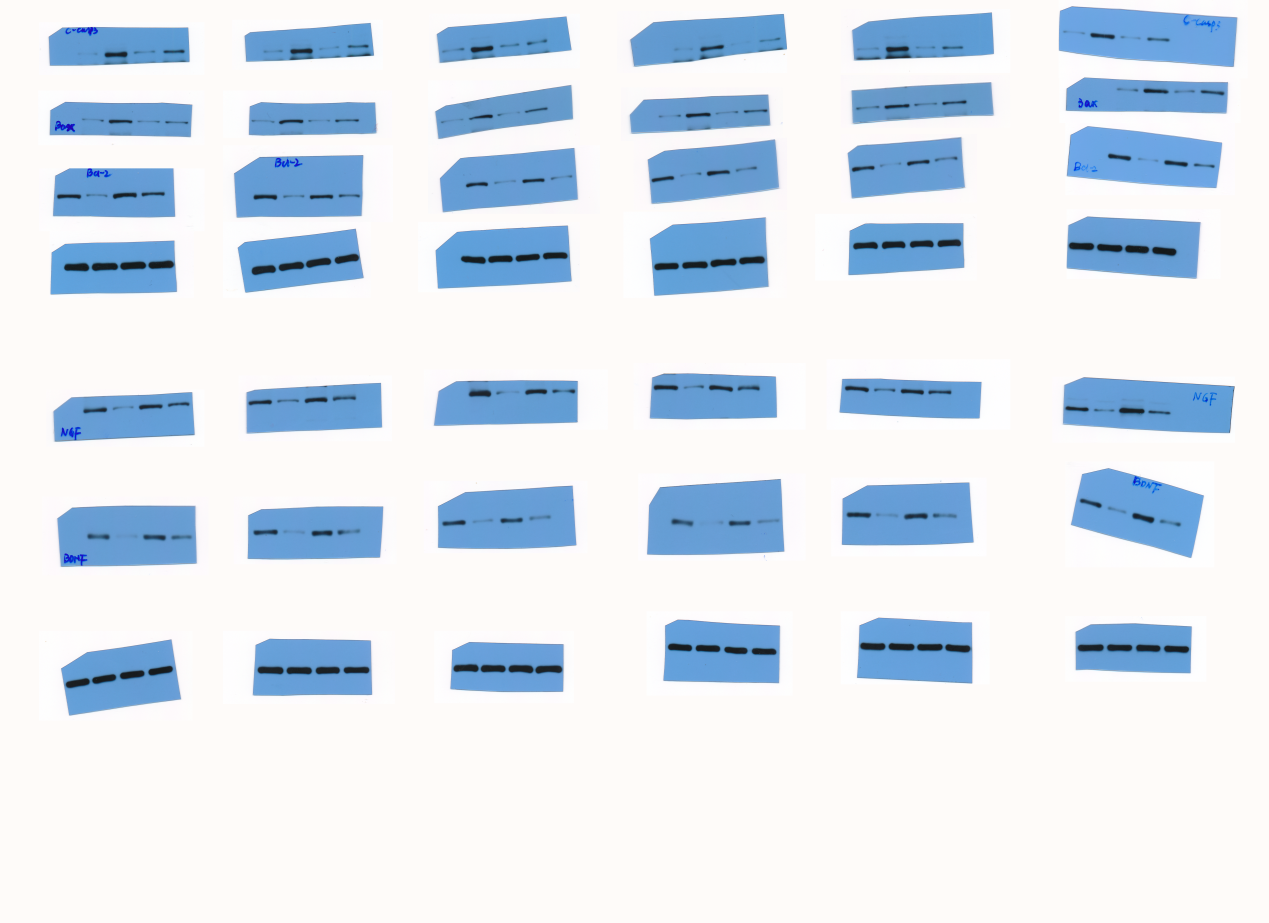
**

**Supplementary Fig 7: original full western blot images**


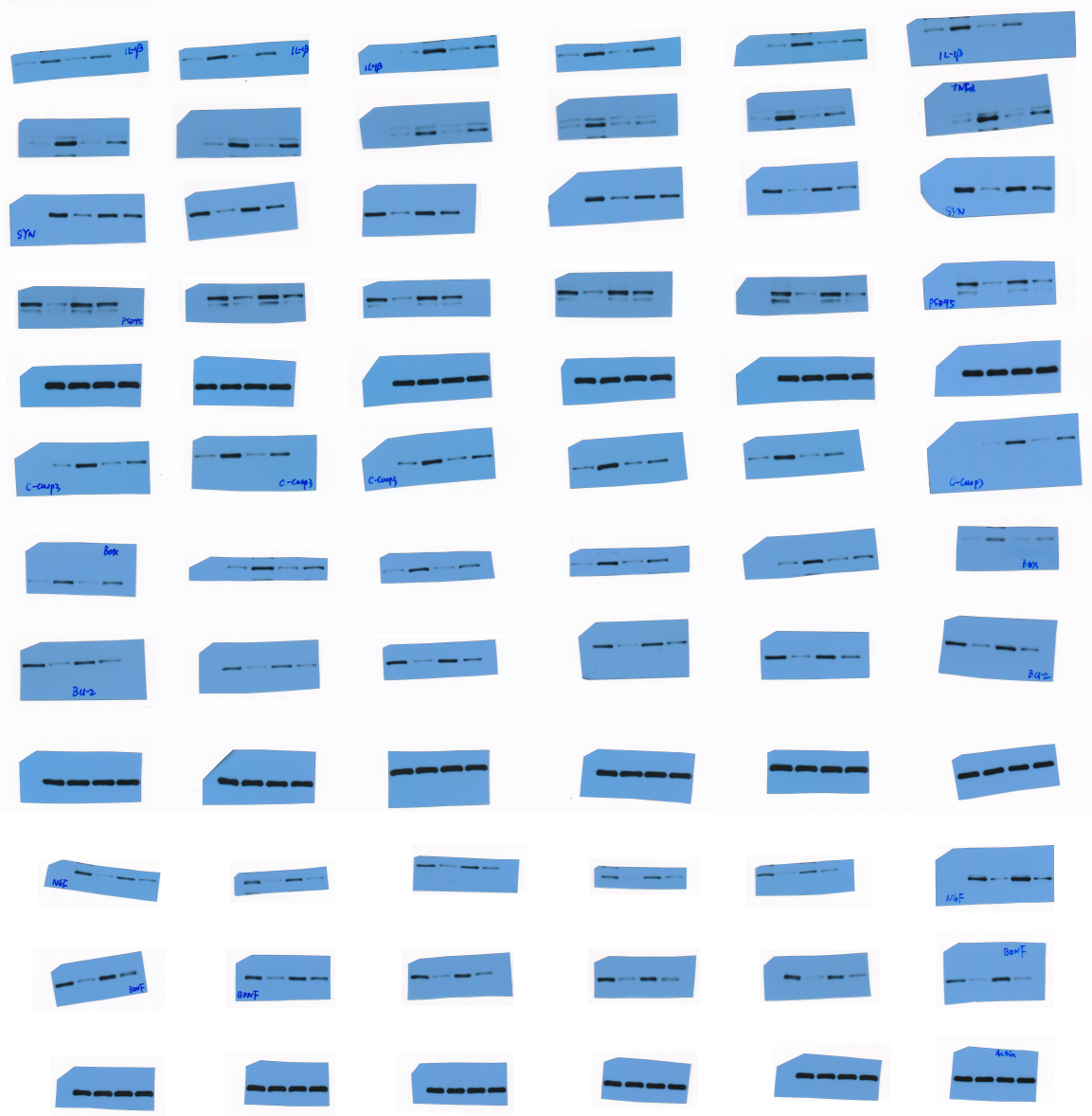


**Supplementary Fig 8: original full western blot images**


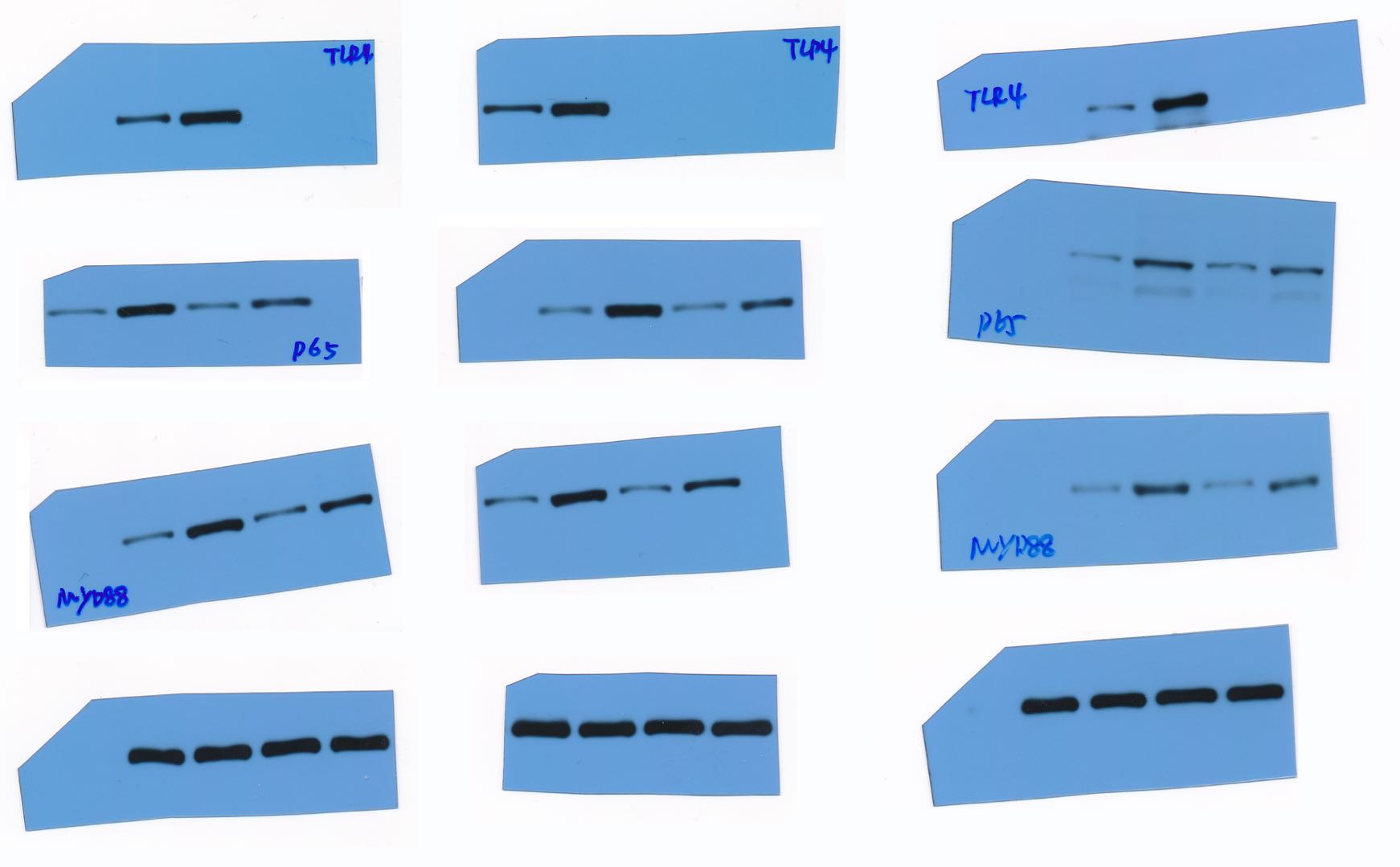

Supplement: Supplementary file 1 — Additional file 1: This document contains additional information on the original full western blot images from all the samples. [file 13041_2020_559_MOESM1_ESM.docx]
